# Transient Absorption Spectroscopic Investigation of the Photocyclization–Deprotection Reaction of 3′,5′-Dimethoxybenzoin Fluoride

**DOI:** 10.3390/molecules29040842

**Published:** 2024-02-14

**Authors:** Runhui Liang, Yuanchun Li, Kin Cheung Lo, Zhiping Yan, Wenjian Tang, Lili Du, David Lee Phillips

**Affiliations:** 1 Tech X Academy, Shenzhen Polytechnic University, Shenzhen 518055, China; rhliang5@szpu.edu.cn; 2 Department of Chemistry, The University of Hong Kong, Hong Kong SAR 999077, China; fionalyc@connect.hku.hk (Y.L.); timlo@connect.hku.hk (K.C.L.); 3 Institute of Advanced Materials, Nanjing Tech University, Nanjing 211816, China; iamzpyan@njtech.edu.cn; 4 School of Pharmacy, Anhui Medical University, Hefei 230032, China; ahmupharm@126.com; 5 School of Life Sciences, Jiangsu University, Zhenjiang 212013, China

**Keywords:** excited-state dynamic, photoremovable protecting group, solvent addition, α-keto cation

## Abstract

The 3′,5′-dimethoxybenzoin (DMB) system has been widely investigated as a photoremovable protecting group (PRPG) for the elimination of various functional groups and has been applied in many fields. The photolysis of DMB fluoride leads to a highly efficient photocyclization–deprotection reaction, resulting in a high yield of 3′,5′-dimethoxybenzofuran (DMBF) in a MeCN solution, while there is a competitive reaction that produces DMB in an aqueous solution. The yield of DMB increased as the volume ratio of water increased. To understand the solvent effect of the photolysis of selected DMB-based compounds, a combination of femtosecond to nanosecond transient absorption spectroscopies (fs-TA and ns-TA), nanosecond time-resolved resonance Raman spectroscopy (ns-TR^3^) and quantum chemical calculation was employed to study the photophysical and photochemical reaction mechanisms of DMB fluoride in different solutions. Facilitated by the bichromophoric nature of DMB fluoride with electron-donating and -withdrawing chromophores, the cyclized intermediates could be found in a pure MeCN solution. The deprotection of a cyclic biradical intermediate results in the simultaneous formation of DMBF and a cyclic cation species. On the other hand, in aqueous solution, fs-TA experiments revealed that α-keto cations could be observed after excitation directly, which could easily produce the DMB through the addition of a hydroxyl within 8.7 ps. This work provides comprehensive photo-deactivation mechanisms of DMB fluoride in MeCN and aqueous conditions and provides critical insights regarding the biomedical application of DMB-based PRPG compounds.

## 1. Introduction

**Scheme 1:**
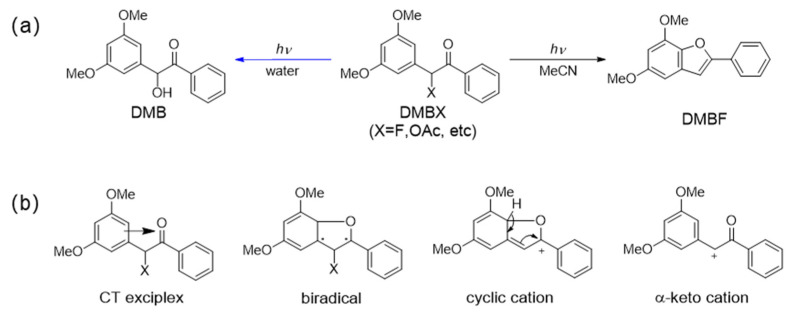
(**a**) The photoreaction of DMBX in different solutions. (**b**) The proposed intermediates involved in the photocyclization–deprotection mechanism.

The 3′,5′-dimethoxybenzoin (DMB) system, as an important member of the photoremovable protecting group (PRPG), has received great research interest and has been developed widely for its efficient photo-releasing functional groups such as nucleotides, carboxylates, inorganic phosphates, alcohols and so on [1,2,3,4,5,6,7]. Moreover, DMB photo-triggers have been applied in different fields, such as drug delivery, DNA synthesis, muscle relaxation, etc. [5,6,8,9,10,11]. The widespread application of the DMB system (DMBX) as a photo-trigger is due to the fact that the photolysis of DMBX is a very clean chemical reaction, providing a photocyclized product, 3′,5′-dimethoxybenzofuran (DMBF), with a high yield (Scheme 1). DMBX, a benzoin derivative distinguished by its two-fold *m*-methoxyl substitution on the benzylic ring, exhibited an impressive photocyclization product yield of 94%, surpassing the yield achieved by benzoin derivatives with a single *m*-methoxyl substitution [4]. The product yield was determined by the number of the substitution group and also the position of methoxyl substitution. For example, under identical conditions, *m*-methoxybenzoin acetate demonstrated a substantial photocyclized product yield of approximately 88%, outperforming *p*-methoxybenzoin acetate, with a yield of 10% [4]. Also, the product yield for *p*-methoxybenzoin acetate was even lower than that of unsubstituted benzoin acetate, with a yield of 20% [12]. The presence of an appropriately situated electron-donating group in benzoin derivatives was able to enhance the effectiveness of photocyclization because of the electrophilic nature of the nπ* carbonyl group. Such a significant difference indicates that the DMB system could be a superior candidate for PRPG in the benzoin family. 

As revealed by the transient absorption techniques, the difference in the photocyclization product yields between benzoin-based PRPG compounds and DMB-based PRPG compounds are mainly due to the differences in the deprotection pathways [13,14]. For benzoin-based PRPG compounds, a direct cyclization–deprotection process occurring within 11 ns in the benzoyl-localized nπ* triplet excited state was verified for benzoin diethyl phosphate in MeCN [13]. For DMB-based PRPG compounds, however, the cyclization–deprotection process was mediated by the singlet excited state and cyclized biradical species, since the DMBF formed while the triplet excited state still existed. This observation was consistent with the fact that triplet state quenchers did not quench the deprotection process of DMB PRPG compounds [4,15]. The photocyclization–deprotection mechanisms of DMBX compounds involving different intermediates and various deactivating paths were summarized, and it was reported that DMBF was produced from the deprotected cyclic cations [14], while direct experimental evidence proved that the precursor of DMBF was lacking. With the help of femtosecond transient absorption spectroscopy (fs-TA) and time-dependent density functional theory (TD-DFT) calculations, our group revealed that the precursor of DMBF was a biradical species for the first time, correcting the previously proposed photocyclization–deprotection mechanism of the DMB-based compounds in MeCN solution [14]. Also, Wirz’s group found that the biradical species were the precursors of the cyclic cations using fs-TA investigation based on DMB acetate [16]. Furthermore, Wan and co-workers suggested that the charge transfer (CT) exciplex could be considered as the precursor of the cyclic intermediate(s) [17], which was demonstrated by our group experimentally and theoretically by studying the electron-rich dimethoxybenzene ring and the electron-deficient acetophenone chromophores [14,18]. The structures of some crucial intermediates are displayed in Scheme 1b. The photocyclization–deprotection reaction of DMB PRPG compounds in MeCN was proven experimentally, but surprisingly this was not the case for DMB chloride, which underwent a chloride exchange reaction in MeCN. This reaction was initiated by the α-keto cations formed by the direct hetero-cleavage of the C-X bond [19]. More importantly, the solvent might affect the photochemical reaction of the benzoin derivatives. For example, the photolysis of benzoin diethyl phosphate in solutions containing a large amount of water mainly produced a hydroxyl addition product, and this was the case for some DMB derivatives (Scheme 1a). It was assumed that the formation of solvent addition products after DMB derivatives’ photolysis was because of the generation of the α-keto cations [13,20]. The consideration of water’s influence was crucial when employing DMBX as a photo-trigger for drug delivery or other biomedical applications, given that the photochemical reaction occurred in aqueous solutions rather than in anhydrous solutions (such as MeCN). Nevertheless, the detailed mechanism of DMBX in aqueous solutions remains unclear.

Time-resolved spectroscopies and TD-DFT calculation are powerful techniques that are widely used to investigate the photophysical and photochemical mechanisms of materials of interest [21,22,23,24,25,26]. Therefore, aiming to investigate the reaction mechanisms of DMB PRPG compounds thoroughly, we employed time-resolved spectroscopies and TD-DFT calculation to study the photochemistry of DMB fluoride in both MeCN and aqueous solutions. The results of this work demonstrate that the solvent addition product was indeed produced from the α-keto cations, but also that the entire photochemical mechanism was different from that observed in other benzoin-based PRPG compounds [13]. To the best of our knowledge, this study marks the first comprehensive exploration offering direct structural characterizations of the intermediates engaged in the photolysis of DMB fluoride. These findings not only enhance our comprehension of the distinctive photochemical behavior exhibited by DMB fluoride in both MeCN and aqueous solutions but also furnish insights that can have broader applications within the field of photoremovable protecting group compounds.

## 2. Results and Discussion

### 2.1. Product Analysis of DMB Fluoride in MeCN and Aqueous Solutions after Photoexcitation 

**Figure 1:**
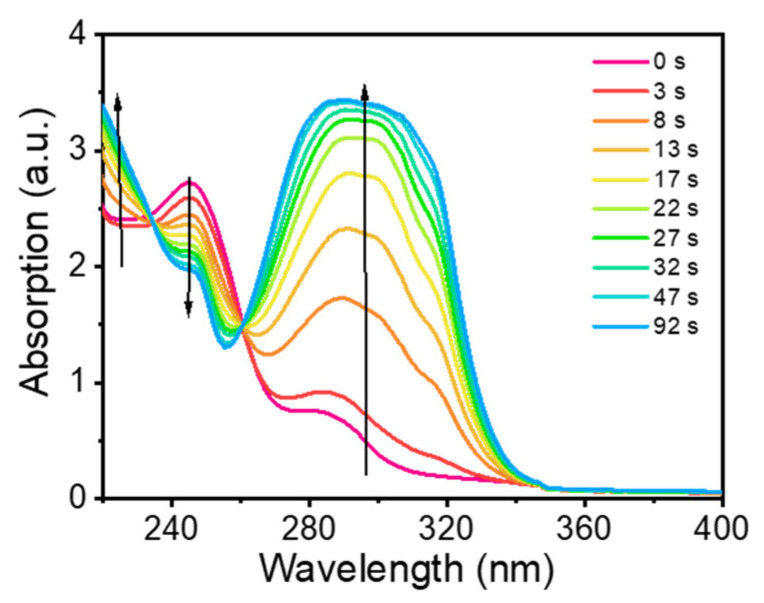
Changes in the absorption spectrum of DMB fluoride in MeCN with an increase in irradiation time (0 s to 92 s) under 266 nm photolysis.

DMB fluoride demonstrated three obvious absorption peaks at 220, 245 and 285 nm in MeCN (0 s spectrum in Figure 1). As shown in Figure 1, after 266 nm monochromatic light photolysis, the UV-vis absorption of DMB fluoride transferred into a different spectrum (the 92 s time spectrum in Figure 1) with a relatively increased absorption band below 220 and at around 300 nm and a decreased absorption band at 245 nm, which was identical to the photocyclized product DMBF reported previously [4,27]. It also matched the calculated absorption spectrum of DMBF with a large oscillator strength at 313 nm (Figure S1a). Although the structure of DMB fluoride was similar to that of DMB chloride, the deprotecting group exchange reaction was not found in DMB fluoride [19]. The well-defined isosbestic points observed at 233 and 260 nm indicated that the transformation of DMB fluoride into DMBF was a direct photo-induced conversion. However, as shown in Figure S2, with the presence of water, the absorption band around 300 nm increased at a much slower rate compared to the results obtained in MeCN, which might be due to the generation of DMBF through photocyclization–deprotection with lower efficiency in aqueous solutions and/or other photochemical reactions (such as the solvent addition reaction) occurring under these conditions. 

To understand the photochemical reactions in neat MeCN and aqueous solutions, a set of product analysis experiments were performed by employing reversed-phase high-performance liquid chromatography (HPLC) to determine the photoproduct distribution. As depicted in Figure S3a, the DMB fluoride underwent a very clean photoconversion to provide DMBF in a neat MeCN solution after photolysis. The quantum yield of this conversion was 0.72 (Table S1), which was larger than the results reported for DMB acetate (0.644) [4] and DMB diethyl phosphate (0.62) [14]. Unsurprisingly, the product’s distribution pattern changed when a different volume ratio of water was added into the MeCN solution after 15 min of UV photolysis (Figure S3b): with an increasing amount of water, the yield of DMBF decreased and another product (DMB) was produced, which could be also accounted for by the different absorption trace patterns displayed in Figure 1 and Figure S2, since the oscillator strength of DMB at 300 nm was much smaller (0.031), as shown in Figure S1b. Apparently, the reaction pathways required to generate DMBF and DMB were competitive in aqueous solutions, determined by the percentage of water. Moreover, the quantum yield of DMBF dropped to 0.56 in the MeCN/water (*v*:*v* = 1:1) mixed solution (Table S1). The quantum yield was not affected by oxygen, which revealed that the triplet intermediate might not be involved in the photolysis of DMB fluoride.

To establish the photochemical process of DMB fluoride after irradiation, an ns-TR^3^ experiment was conducted to provide the “fingerprint” information, as shown in Figure S4. DMB fluoride in neat MeCN and an aqueous solution was excited with a 266 nm laser and probed with a 309 nm laser. Upon the excitation of DMB fluoride, the characteristic Raman peaks at 1610, 1565 and 994 cm^−1^ could be observed with a delay of 5 ns (time resolution of our ns-TR^3^ technique) in both the neat MeCN and aqueous solutions, which could be assigned to the cyclized product DMBF [14]. Moreover, the TD-DFT calculation-predicted Raman vibrations of DMBF were in good agreement with the experimental results (Figure S5). The absence of the DMB signals might be due to its weak absorption at 309 nm. These results also indicate that the DMBF was produced within 5 ns.

### 2.2. Transient Absorption Spectroscopic Investigation of DMB Fluoride in Neat MeCN and Aqueous Solutions

**Figure 2:**
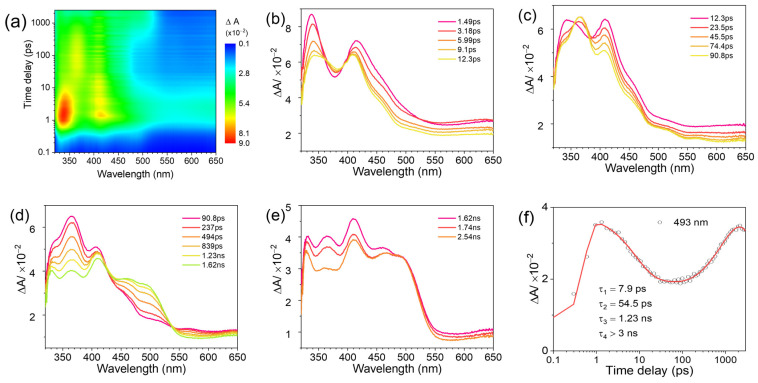
Fs-TA of DMB fluoride in a neat MeCN solution upon 267 nm photoexcitation. (**a**) Three-dimensional contour plot; (**b**–**e**) spectral changes at different time delays; (**f**) kinetics of the characteristic absorption band observed at 493 nm.

The photophysical and photochemical dynamics of DMB fluoride after 267 nm laser photolysis in a neat MeCN solution were recorded by employing fs-TA. Four characteristic temporal evolutions regarding the changes in transient absorption peaks at around 338, 366, 408 and 500 nm can be clearly seen in Figure 2a. Upon 267 nm irradiation, the DMB fluoride is excited to the upper singlet excited state (S_n_) and a prompt internal conversion (IC) at the sub-picosecond time scale is expected to produce the lowest nπ* singlet excited state (S_1_) with transient absorption bands at 338 and 414 nm (1.49 ps spectrum in Figure 2b). From 1.49 to 12.3 ps, the 338 nm peak decays rapidly, and the 414 nm peak blue-shifts to 408 nm with lower absorption intensity. Two isosbestic points at 360 and 394 nm can be clearly seen, indicating a straightforward conversion from the S_1_ excited state to other intermediates. Once the absorption band at 408 nm forms, it decays gradually until reaching the detection limit of our fs-TA instrument (3 ns), and it is predicted as the absorption of the triplet excited state of the DMB fluoride, confirmed by the ns-TA experiment discussed later. As mentioned previously, the cyclized product is not produced through the triplet excited state, so there must be another deactivated channel for the S_1_ excited state. According to the previous results for DMB diethyl phosphate [14], the band at 360 nm could be due to the CT exciplex, which is likely produced from the S_1_ excited state and becomes the precursor in the subsequent cyclization and deprotection reactions. Then, the decay of the CT exciplex absorption band gives rise to a sharp absorption band at 366 nm until 90.8 ps (Figure 2c), which could be assigned to the biradical absorption reported for DMB diethyl phosphate [14,18]. Therefore, this process comprises the cyclization reaction forming a five-member ring with a biradical character generated from the radical-based addition of excited carbonyl oxygen to the substituted benzylic ring [3]. From 90.8 ps to 1.62 ns, as shown in Figure 2d, the intense absorption band at 366 nm starts to become depopulated, accompanied by the growth of a broad absorption band at 500 nm, displaying a shallow peak at 330 nm (Figure 2d). Finally, all of these peaks decrease at different rates individually (Figure 2e). The characteristic absorption band appearing at 500 nm is attributed to the cyclic cation species with a long lifetime of 0.13 μs for DMB acetate [17]. To support the assignment of the cyclic cations, the calculated absorption spectrum of the cyclic cations revealed by means of TD-DFT calculation was perfectly matched with the experimental data (Figure S1c and Figure 2d). Although the calculated absorption spectrum of the cyclic cations presents an absorption band below 300 nm as well, it is not appropriate to assign the absorption band at 330 nm to the cyclic cations only, because the band at 330 nm barely decays, while the 500 nm band declines gradually, as shown in the ns-TA results (Figure S6). Therefore, another species may be generated from the biradical intermediate and contribute to the 330 nm band. For its stability in ns-TA, this could easily be assigned to the photocyclization–deprotection product DMBF [14,28], and this was consistent with the results demonstrated in Figure 1 and Figure S6. Thus, the defluorination of the biradical species could occur through E1 elimination to provide cyclic cations and E2 elimination to produce DMBF competitively. The oxygen quenching experiment was also employed to predict the previous assignment of the 408 nm band. As demonstrated in Figure S6b, the lifetime of the 408 nm band in a deoxygenated MeCN solution (1.35 μs) is much longer than that under open air conditions (0.13 μs), which further confirms that the 408 nm band is naturally a triplet excited state species. 

**Scheme 2:**
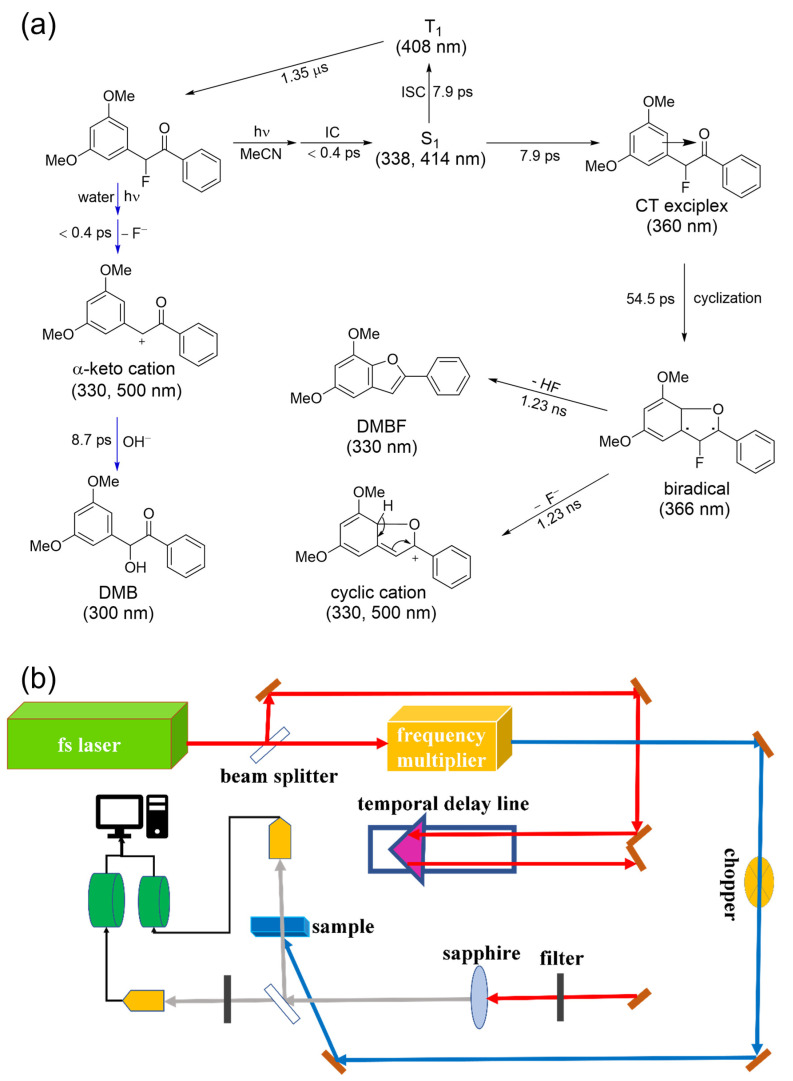
(**a**) The proposed mechanism of DMB fluoride after 267 nm laser photolysis; (**b**) experimental set-up of fs-TA.

Therefore, the photochemical process of DMB fluoride in a MeCN solution was relatively clear, and the characteristic absorption band at 493 nm was fitted by using an exponential function, providing four time constants, namely 7.9 ps, 54.5 ps, 1.23 ns and >3 ns (Figure 2f). The proposed photocyclization–deprotection reaction of DMB fluoride in a MeCN solution is displayed in Scheme 2. After photoexcitation, a very fast IC process occurs within the instrumental response, and the S_1_ excited state is born. Two competing deactivation processes for the S_1_ excited state can be observed: (1) the ISC process forming the triplet excited state, which decays into the ground state through another ISC process [17]; and (2) the generation of the CT exciplex in 7.9 ps (the decay time constant of the S_1_ excited state). The short lifetime of the S_1_ excited state compared to the exciplex indicates a barrierless excited-state conversion process. The cyclization process takes place from the CT exciplex precursor to form the biradical species in 54.5 ps, and the fluoride ion is deprotected to produce either cyclic cations or the final product DMBF in around 1.23 ns. While these reaction pathways closely resemble the photolysis of DMB diethyl phosphate previously reported by our group [14], the photodynamics of DMB fluoride in a MeCN solution served as a valuable reference when analyzing the fs-TA results obtained in aqueous solutions.

**Figure 3:**
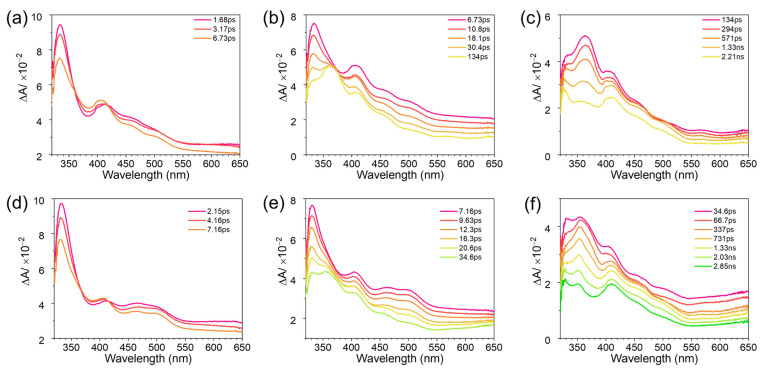
(**a**–**c**) Fs-TA spectral changes of DMB fluoride in MeCN:H_2_O (4:1, *v*/*v*) solution upon 267 nm photoexcitation at different time delays. (**d**–**f**) fs-TA spectral changes of DMB fluoride in MeCN:H_2_O (1:1, *v*/*v*) solution upon 267 nm photoexcitation at different time delays.

**Figure 4:**
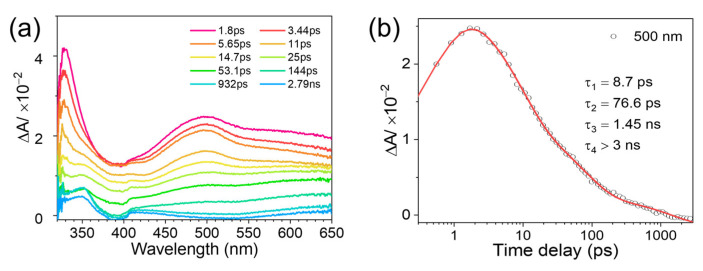
Fs-TA of DMB fluoride in MeCN:H_2_O (1:4, *v*/*v*) solution upon 267 nm photoexcitation. (**a**) Spectral changes at different time delays; (**b**) kinetics of the characteristic absorption bands observed at 500 nm.

With the addition of water, the photodynamics of the DMB fluoride changed, since there competitive reactions occurred. The fs-TA experiments were conducted on the DMB fluoride in mixed MeCN and H_2_O solutions (volume ratio 4:1, 1:1 and 1:4), and the results are depicted in Figure 3 and Figure 4. Overall, the signal of the biradical intermediate detected in neat MeCN solution decreased, while new species at 330 and 500 nm could be observed as the volume ratio of H_2_O increased. The TA signals of DMB fluoride in MeCN:H_2_O (4:1 and 1:1) mixed solutions shown in Figure 3 were mostly contributed to by the intermediates taking part in the photocyclization–deprotection reaction. However, the increasing transient absorption at 500 nm assigned to the cyclic cations shown in Figure 2d was barely visible. Very interestingly, the TA peak at 500 nm could be found at around 2 ps (Figure 3a,d), and the relative intensity of this peak was higher as the water content in the solution increased. Therefore, a new species peak occurred at 500 nm in the aqueous solution.

Figure 4 shows the differing photochemical behavior of DMB fluoride in a higher-water-content (80%) solution. Broad transient absorption at 500 nm and a sharp band at 330 nm were produced upon photoexcitation, and their relative intensity was higher than those in 20% and 50% water content solutions. Obviously, this newly found intermediate was favored in aqueous solutions and was associated with the generation of DMB. The α-keto cations were predicted to be the precursors of DMB in aqueous solutions [16]. The calculated absorption of the α-keto cations based on TD-DFT matched the experimental data well (Figure S1d). Thus, the 500 nm peak with low intensity observed in 20% and 50% water content solutions could be attributed to the appearance of the α-keto cations in aqueous solutions as well. Then, this newly found intermediate decayed quickly to produce DMB by eliminating the hydroxyl group from water. After the decay of the α-keto cations, some remaining peaks present at the later delay times were found to be reasonably in line with the intermediates appearing in a neat MeCN solutions. The kinetics of the characteristic absorption band at 500 nm were fitted by employing an exponential function and the time constants were 8.7 ps, 76.6 ps, 1.45 ns and >3 ns (Figure 4b). Together with the spectral analysis, the 8.7 ps time constant could be reasonably assigned to the decay of the α-keto cations, while their generation lifetime was unable to be obtained due to the instrumental limit (400 fs). Consistent with the photochemical reaction in neat MeCN solution, the other time constants (76.6 ps, 1.45 ns and >3 ns) corresponded to the decay of the CT exciplex, biradical intermediate and cyclic cations, respectively. Therefore, the photodynamics of DMB fluoride in aqueous solutions to produce DMB can be fully explained and are depicted in Scheme 2. Upon photoexcitation, the defluorination of DMB fluoride can easily occur and produces the α-keto cations within a sub-picosecond time range, and the α-keto cations are not a stable intermediate, producing DMB via hydroxyl addition within 8.7 ps. 

## 3. Materials and Methods

### 3.1. Synthesis and Characterization

DMB Fluoride: 3′,5′-dimethoxybenzoin (270 mg, 1.0 mmol), perfluoro-1-butanesulfonyl fluoride (PBSF) (1.4 mL, 8.0 mmol), NEt_3_(HF)_3_ (1.3 mL, 8.0 mmol), and *i*-Pr_2_NEt (3.0 mL, 20 mmol) in THF (10 mL) were stirred overnight at room temperature. After TLC was used to detect the end of the reaction, EtOAc (50 mL) was added, and the mixture was washed with water twice. The organic layer was separated and dried with Na_2_SO_4_, filtrated, and concentrated in vacuo. The residue was purified by means of flash chromatography (silica gel-*H*, petroleum ether/EtOAc) to obtain DMB fluoride as a colorless oil (190 mg, 70%). ^1^H-NMR (300 MHz, CDCl_3_): *δ* = 3.77 (s, 6 H, OCH_3_), 6.33, 6.49 (d, coupling with F, 1 H, FCH), 6.42 (m, 1 H, Ar-H), 6.62 (m, 2 H, Ar-H), 7.43 (m, 2 H, Ar-H), 7.56 (m, 1 H, Ar-H), 7.95 (m, 2 H, Ar-H); HRMS (ESI-TOF) calcd. for C_16_H_15_FNaO_3_ [M + Na]^+^ 297.0897, found 297.0898.

DMBF: DMB acetate (120 mg, 0.38 mmol) was dissolved in MeCN (100 mL). The solution was bubbled with high-purity nitrogen for 20 min, and then degassed for 15 min in an ultrasonic bath. The solution was irradiated for 30 min with a 300 W high-pressure Hg lamp in a Pyrex photochemical reactor. The reaction mixture was concentrated in vacuo. The residue was dissolved in dichloromethane and subjected to column chromatography (silica gel-*H*, petroleum ether/EtOAc) to yield the photoproduct DMBF as a white powder (78 mg, 80%). M.p. 82–83 °C; ^1^H-NMR (300 MHz, CDCl_3_): *δ* = 3.85 (s, 3 H, OCH_3_), 4.01 (s, 3 H, OCH_3_), 6.45 (d, J = 2.2 Hz, 1 H, benzofuran-H), 6.62 (d, J = 2.2 Hz, 1 H, benzofuran-H), 6.95 (s, 1 H, furan-H), (7.33 (m, 1 H, Ar-H), (7.43 (m, 2 H, Ar-H), (7.87 (m, 2 H, Ar-H); HRMS (ESI-TOF) calcd. for C_16_H_15_O_3_ [M + H]^+^ 255.1016, found 255.1020.

### 3.2. Experimental

Setup of fs-TA. A general description of this setup and the related methods is given here, while details can be found in our previous study [29]. The experimental setup is shown in Scheme 2b. The amplified laser pulses (800 nm) were seeded from a femtosecond regenerative amplified Ti:sapphire laser system and split into a pump laser beam and a probe laser beam for use in the fs-TA experiments. The probe laser pulse was produced by utilizing ~5% of the amplified 800 nm laser pulses to generate a white-light continuum (325−650 nm) in a CaF_2_ crystal and the pump beam was adjusted to 267 nm. The maximum temporal delay line in this instrument was 3 ns. All sample solutions (50 mL) with an absorbance of 1 at 267 nm were excited in a flowing 2 mm path-length quartz cuvette. The ratios of water and ACN indicated in the article were the volume ratios.

Setup of ns-TA. Based on the detailed experimental setup described in [29], ns-TA experiments were conducted on a LP920 laser flash spectrometer obtained from Edinburgh Instruments Ltd. The probe light was a 450 W ozone-free Xe arc lamp with a continuous spectrum from 280 to 800 nm, and the 266 nm pump beam was obtained from the 4th harmonic output of a Nd:YAG laser. The samples in the 1 cm path-length quartz cuvette were excited by the pump laser prior to being detected by the probe light and the transited probe light was collected by detectors for further investigations. Sample solutions were freshly prepared with an absorbance of about 1 at 266 nm in a 1 cm path-length quartz cuvette.

Setup of ns-TR^3^**.** The ns-TR^3^ experiments were performed using an experimental apparatus and methods discussed in detail in [29,30]. The pump laser pulse with a wavelength of 266 nm generated from the fourth harmonic of a Nd:YAG nanosecond pulsed laser and a 309 nm probe laser pulse produced from the first anti-Stokes hydrogen Raman-shifted laser line from the third harmonic were employed in the TR^3^ experiments. The two Nd:YAG lasers were synchronized electronically by a pulse delay generator to control the time delay of the pump and probe lasers, and the time delay between the laser pulses was monitored by means of a fast photodiode and a 500 MHz oscilloscope. The time resolution for the TR^3^ experiments was approximately 10 ns. The pump and probe laser beams were focused on the sampling system, and the Raman scattering signal was collected using reflective optics in a spectrometer whose grating dispersed the light onto a liquid nitrogen-cooled CCD detector. The Raman signal was acquired for 20 s by the CCD before being displayed on the computer, and 10 scans of the signal were accumulated to produce a resonance Raman spectrum. The TR^3^ spectra presented here were obtained via the subtraction of a resonance Raman spectrum with a negative time delay of −100 ns from the resonance Raman spectrum. The TR^3^ spectra in this work were calibrated using the known MeCN solvent Raman bands with an estimated accuracy of 5 cm^−1^. Samples of the DMB fluoride solutions were prepared to have an absorption of 1 at 266 nm in a 2 mm path-length cuvette and then were used in the TR^3^ experiments.

DFT/TD-DFT Calculations. The geometry optimization and vibrational frequency analysis were performed by employing the B3LYP method and the 6-311G** basis set with the CPCM solvation model of MeCN for the DMBF and cyclic cations, and water for the DMB and α-keto cations, respectively. The calculated Raman spectra were scaled with a factor of 0.98. The calculated absorption spectra of DMBF, DMB, cyclic cations and α-keto cations were obtained using TD-DFT calculations and the electronic absorption spectra were estimated using GaussSum software 3.0 using a half band width of 2500 cm^−1^ [31]. All of the calculations were achieved using the Gaussian16 software (Revision C.01) suite installed on a high-performance computing cluster at the University of Hong Kong [32].

Measurements of Quantum Yields of Photochemistry of DMB Fluoride: To measure the quantum yields of the photocyclization–deprotection of DMB fluoride, [Φ = (rate of photocyclization–deprotection)/(rate of photons absorbed)], all of the sample solutions (~5 × 10^−5^ M, 3 mL) were prepared with the corresponding solvents and placed in quartz cuvettes with a Teflon stopper, then irradiated with 320 nm wavelength light from a fluorescence spectrometer operated with a 10 nm slit. The absorbance wavelengths at 300 nm (*A*_300_) and 320 nm (*A*_ex_) were recorded at certain intervals of time after irradiation. The extent of the photocyclization–deprotection was measured by monitoring the increase in the absorbance at 300 nm (*A*_300_) due to the regeneration of the cyclized product DMBF. The *A*_300_ change (Δ*A*_300_) of the solution depended on the extent of the photocyclization–deprotection. The plot of Δ*A*_300_ against the irradiation time (*t*, min) fitted to a straight line well, where the slope of the straight line *B* reflected the photo-reacting rate of DMB fluoride. The intensity of the excitation light beam (*I*_0_, unit: einstein min^−1^) was measured through ferrioxalate actinometry. The intensity of light absorbed (*I*_a_) by the solution was calculated in terms of Beer’s law, *I*_a_ = *I*_0_(1 − 10^−Aex^). The change in the mole extinction coefficients (Δ*ε*_300_) was obtained from the UV absorption spectra of DMB fluoride and the cyclized product DMBF. These values allowed the calculation of the quantum yield, Φ = *BV*_0_/Δ*ε*_300_*I*_a_, wherein *V*_0_ is the volume of irradiation solution, 3 × 10^−3^ L, and the experimental error is within 10%. The quantum yields of photocyclization–deprotection did not significantly change with and without N_2_ bubbling prior to irradiation. Hence, the non-deaerated solution was applied for all of the measurements of the quantum yield. To limit the competition for the absorption of the irradiated light between test compounds and the photoproduct, the extent of the photocyclization–deprotection of test compounds was controlled within 10% in all of the measurements of the quantum yield.

## 4. Conclusions

A systematic investigation into the photochemistry of DMB fluoride in MeCN and aqueous solutions was achieved by employing TA spectroscopies and steady-state experiments. The steady-state experiments illustrated a distinct difference in photolysis pathways, with DMB fluoride in a neat MeCN solution undergoing a clean and efficient conversion to DMBF, while aqueous solutions predominantly displayed a hydroxyl addition reaction, leading to the formation of a DMB product. The time-resolved experiments were utilized to further elucidate the underlying mechanisms. In neat MeCN, the formation of a biradical intermediate was facilitated by the charge-transfer (CT) exciplex, and later the biradical intermediate resulted in the simultaneous production of DMBF and cyclic cations by means of defluorination within 1.23 ns. The introduction of water brought about an alternative route for DMB fluoride photolysis in an aqueous solution. Here, the α-keto cations emerged directly within sub-picoseconds, and they subsequently transformed into DMB through hydroxyl addition within 8.7 ps. These findings underscore the nuanced photolysis processes of DMB fluoride in diverse solvent environments. Significantly, this study highlights the pivotal role of solvent choice in reaction pathways, offering valuable insights for future research endeavors and practical applications, especially biomedical usages in the field of PRPG molecules.

## Data Availability

Data are contained within the article and Supplementary Materials.

## Supplementary Materials

The following supporting information can be downloaded at: https://www.mdpi.com/article/10.3390/molecules29040842/s1. Figure S1: The calculated absorption spectra of (a) DMBF (in MeCN); (b) DMB (in water); (c) cyclic cations (in MeCN) and (d) α-keto cations (in water) using TD-B3LYP/6-311G(d,p). Figure S2: Change in the absorption spectrum of DMB fluoride in MeCN:water 1:2 (*v*:*v*) with an increase in irradiation time (0 s to 12.5 min) under 266 nm photolysis. Figure S3: (a) Typical HPLC chromatograms obtained after 0, 3, 6, 9, 12 and 15 min of irradiation of DMB fluoride in MeCN (3 mL, 5 × 10^−5^ M) with 320 nm light. Assay conditions: C_18_ reverse-phase column (4.6 mm × 150 mm × 5 μm), mobile phase (MeCN:water = 40:60), detection at 254 nm. Retention times: 4.4 min (DMB fluoride), 7.6 min (photo-product DMBF). (b) Typical HPLC chromatograms obtained after 15 min of irradiation of DMB fluoride in different solvents (MeCN: water, 3 mL, 5 × 10^−5^ M) with 320 nm light. Assay conditions: C_18_ reverse-phase column (4.6 mm × 250 mm × 5 μm), mobile phase (MeCN:water = 60:40), detection at 254 nm. Retention times: 3.3 min (photo-product DMB), 4.7 min (DMB fluoride), 9.0 min (photo-product DMBF). Figure S4: Ns-TR^3^ spectra of DMB fluoride in (a) neat MeCN and (b) MeCN:H_2_O (1:1, *v*/*v*) solutions obtained upon photoexcitation using 266 nm laser light and probing with 309 nm laser light at different delay times. Asterisks (*) mark the regions affected by solvent subtraction. Figure S5: The calculated Raman spectra of DMB and DMBF using B3LYP/6-311G(d,p). Figure S6: (a) Ns-TA spectra of DMB fluoride in a MeCN solution upon 266 nm photoexcitation at vary time delays. (b) The kinetics of the characteristic absorption band observed at 408 nm in an argon-saturated MeCN solution and open-air MeCN solution. The Cartesian coordinates of DMBF, DMB, cyclic cations and α-keto cations are shown. Table S1: The quantum yield (Φ) of DMBF from DMB fluoride in various solutions.
