# Supplementary material for: Transient Absorption Spectroscopic Investigation of the Photocyclization–Deprotection Reaction of 3′,5′-Dimethoxybenzoin Fluoride
Source: Molecules. 2024 Feb 14;29(4):842. doi: 10.3390/molecules29040842 (PMC10892591; doi:10.3390/molecules29040842)
Supplement: Supplementary file 1 [file molecules-29-00842-s001.zip › molecules-2839810-supplementary.pdf]

Supplementary Materials

Transient Absorption Spectroscopic Investigation of the Photocyclization–Deprotection Reaction of 3′,5′-Dimethoxybenzoin Fluoride

Runhui Liang [1,2,†], Yuanchun Li [2,†], Kin Cheung Lo [2], Zhiping Yan [3], Wenjian Tang [4], Lili Du [2,5,*] and David Lee Phillips [2,*]

[1] Tech X Academy, Shenzhen Polytechnic University, Shenzhen 518055, China; rhliang5@szpu.edu.cn
[2] Department of Chemistry, The University of Hong Kong, Hong Kong SAR 999077, China; fionalyc@connect.hku.hk (Y.L.); timlo@connect.hku.hk (K.C.L.)
[3] Institute of Advanced Materials, Nanjing Tech University, Nanjing 211816, China; iamzpyan@njtech.edu.cn
[4] School of Pharmacy, Anhui Medical University, Hefei 230032, China; ahmupharm@126.com
[5] School of Life Sciences, Jiangsu University, Zhenjiang 212013, China
* Correspondence: justlily@connect.hku.hk (L.D.); phillips@hku.hk (D.L.P.)
† These authors contributed equally to this work.

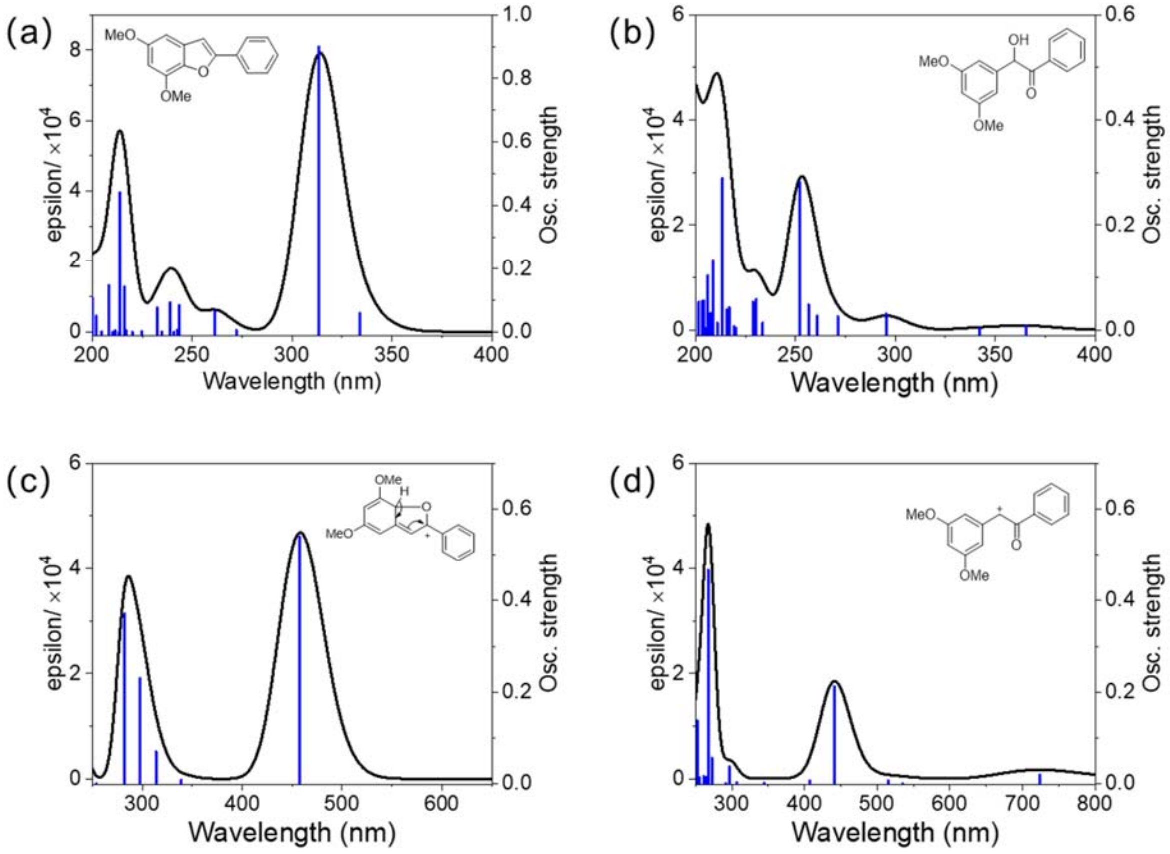

Figure S1. The calculated absorption spectra of (a) DMBF (in MeCN); (b) DMB (in water); (c)cyclic cation (in MeCN) and (d) α-keto cation (in water) using TD-B3LYP/6-311G(d,p).

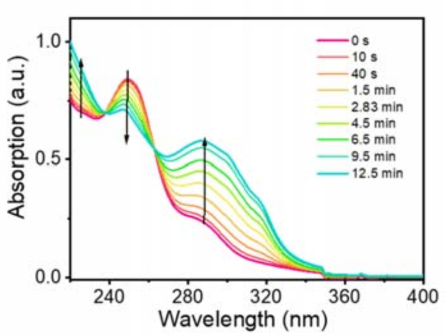

Figure S2. Change in the absorption spectrum of DMB Fluoride in MeCN:water 1:2 (v:v) with increasing of irradiation time (0 s to 12.5 min) under 266 nm photolysis.

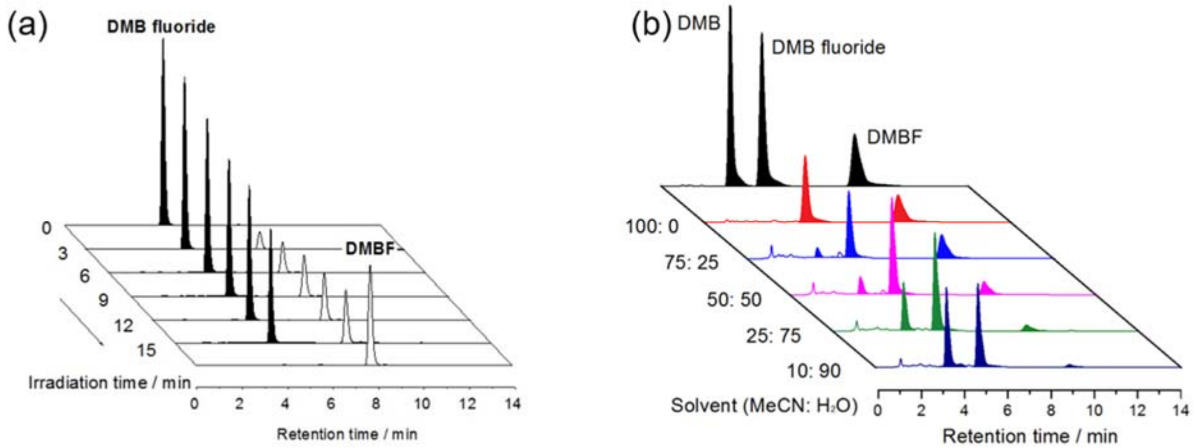

Figure S3. (a) Typical HPLC chromatograms obtained after 0, 3, 6, 9, 12 and 15 min of irradiation of DMB fluoride in MeCN (3 mL, 5×10^{-5} M) with 320 nm light. Assay conditions: C_{18} reverse-phase column (4.6 mm × 150 mm × 5 μm), mobile phase (MeCN: water = 40: 60), detection at 254 nm. Retention times: 4.4 min (DMB fluoride), 7.6 min (photo-product DMBF). (b) Typical HPLC chromatograms obtained after 15 min of irradiation of DMB fluoride in different solvents (MeCN: water, 3 mL, 5×10^{-5} M) with 320 nm light. Assay conditions: C_{18} reverse-phase column (4.6 mm × 250 mm × 5 μm), mobile phase (MeCN: water = 60: 40), detection at 254 nm. Retention times: 3.3 min (photo-product DMB), 4.7 min (DMB fluoride), 9.0 min (photo-product DMBF).

Table S1. The quantum yield (Φ)[a] of DMBF of DMB Fluoride in various solutions.

Solvents	DMB Fluoride
MeCN	0.72
MeCN/water 1:1 (v:v)	0.56

[a] Average of two determinations, experimental error in ±5%.

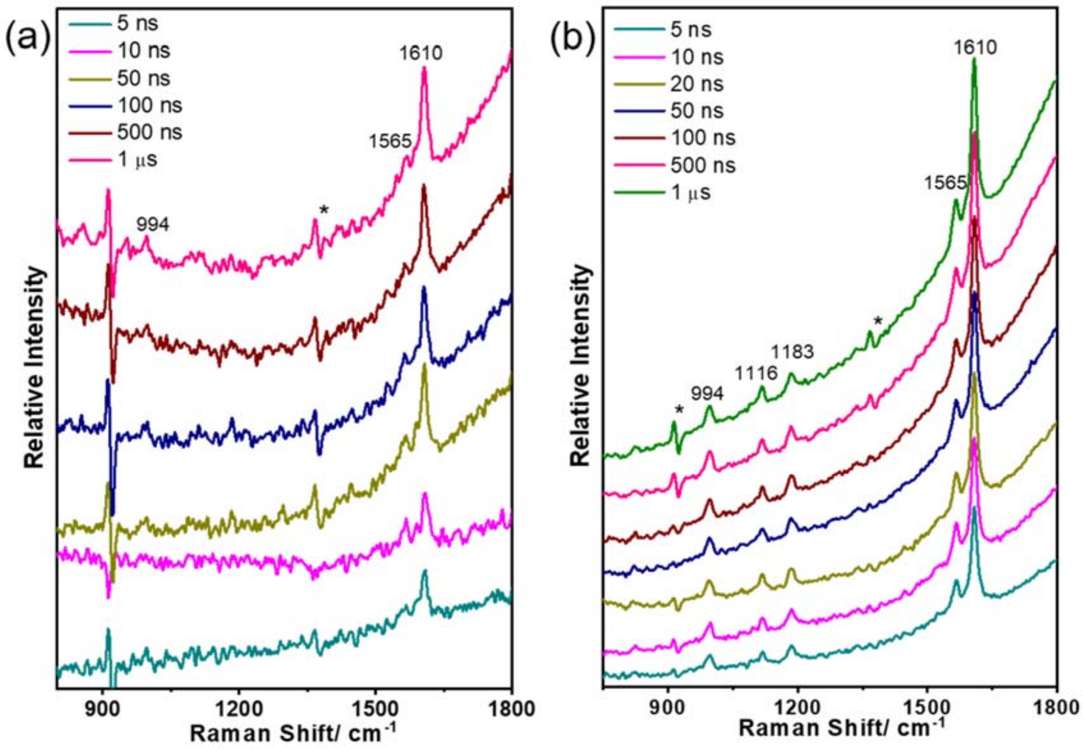

Figure S4. ns-TR[3] spectra of DMB Fluoride in (a) neat MeCN and (b) MeCN:H_2O (1:1, v/v) solutions obtained upon photoexcitation using 266 nm laser light and probing with 309 nm laser light at different delay times. Asterisk (*) marked the regions that affected by solvent subtraction.

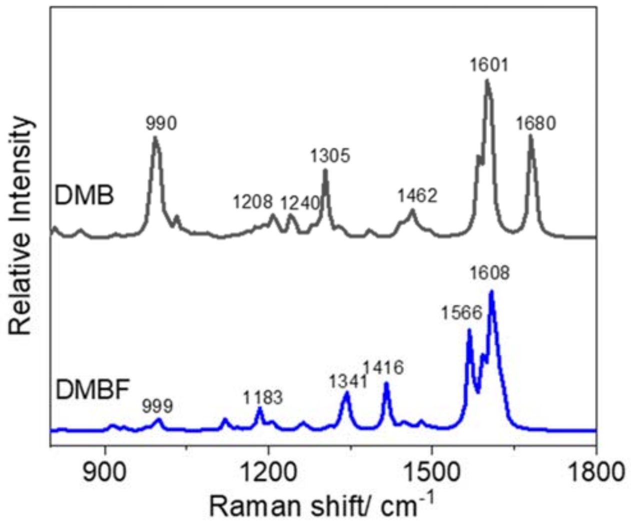

Figure S5. The calculated Raman spectra of DMB and DMBF using B3LYP/6-311G(d,p).

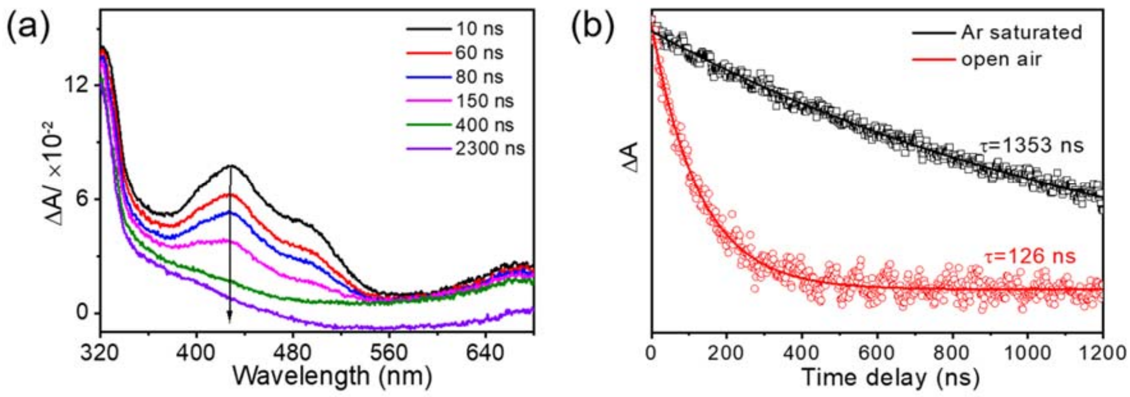

Figure S6. (a)ns-TA spectra of DMB Fluoride in MeCN solution upon 266 nm photoexcitation at vary time delays. (b)The kinetics of the characteristic absorption band observed at 408 nm under Argon saturated MeCN solution and open air MeCN solution.

Cartesian Coordinates:

DMBF, solvent=MeCN
0 1

C	3.09366000	-1.02271900	-0.00020800
C	3.02597300	0.38824700	-0.00132500
C	1.78993600	1.04285800	-0.00171800
C	0.65082100	0.23965200	-0.00110700
C	0.70795000	-1.15890300	-0.00011700
C	1.94739200	-1.81289700	0.00043000
O	-0.64902400	0.67260600	-0.00113000
C	-1.43791100	-0.46067000	-0.00020100
C	-0.66597800	-1.58743300	0.00051500
C	-2.88182800	-0.24836900	0.00010500
O	1.61039300	2.38934000	-0.00274000
O	4.28379300	-1.70279900	-0.00017300
C	-3.41961300	1.04930300	0.00507500
C	-4.79892700	1.24238400	0.00564600
C	-5.66441800	0.14896700	0.00130100
C	-5.13834000	-1.14503200	-0.00370100
C	-3.76240800	-1.34432600	-0.00441000
C	5.50754400	-0.96769500	0.00491900
C	2.76776600	3.23200100	-0.00251500
H	3.93368400	0.97076800	-0.00207400
H	2.03109700	-2.89203500	0.00139100
H	-1.02304300	-2.60473900	0.00154200
H	-2.75488300	1.90349200	0.00859000
H	-5.19734600	2.25057900	0.00954600
H	-6.73747700	0.30134100	0.00176000
H	-5.80268900	-2.00158100	-0.00723800
H	-3.37130200	-2.35471700	-0.00862600
H	5.59486000	-0.34510500	0.90034500
H	5.60160700	-0.34369700	-0.88885200
H	6.29954500	-1.71418300	0.00722100
H	3.37366100	3.06649400	-0.89737600
H	3.37317500	3.06666200	0.89270300
H	2.38809100	4.25134500	-0.00272700

DMB, solvent=water

0 1

C	2.50341300	-1.08847000	0.15933500
C	2.94350400	0.13648800	-0.35175700
C	2.15511200	1.27465600	-0.14057400
C	0.94938300	1.19094900	0.55936700
C	0.52244700	-0.04215700	1.05625300
C	1.29788800	-1.18265700	0.86360000
O	3.19498900	-2.25507100	0.01902400
O	2.49218000	2.51684200	-0.59071700
C	-0.77955700	-0.12183500	1.85443000
C	-1.92183100	0.71062800	1.22546100
C	-2.68786400	0.17217200	0.06188000
C	-4.00843500	0.61016700	-0.12136600
C	-4.76278100	0.14340200	-1.19135700
C	-4.20238400	-0.75540200	-2.10192600
C	-2.88702800	-1.18727900	-1.93591000
C	-2.13330100	-0.73291400	-0.85590200
O	-1.20941800	-1.44615300	2.14912100
O	-2.20867500	1.78516200	1.72646700
C	4.44079100	-2.24241000	-0.68311500
C	3.71303600	2.68474400	-1.31637500
H	3.87218900	0.20436000	-0.89575500
H	0.36974700	2.09403500	0.70533500
H	0.99492700	-2.14343000	1.25736700
H	-0.61078900	0.33712900	2.83083700
H	-4.43329300	1.30914700	0.58847100
H	-5.78578500	0.47796700	-1.31793600
H	-4.78936300	-1.11595600	-2.93879800
H	-2.44568000	-1.87454800	-2.64790100
H	-1.10395800	-1.05712400	-0.75967100
H	-1.40008700	-1.92257900	1.33160900
H	4.80047200	-3.26904400	-0.66281200
H	4.30609800	-1.92422200	-1.72071000
H	5.16704900	-1.59214400	-0.18747000
H	3.76475000	3.74293300	-1.56405600
H	4.57722100	2.41146200	-0.70447900
H	3.70953800	2.09529400	-2.23747400

cyclic cation, solvent=MeCN

1 1

C	-2.64698400	3.25856100	-0.24825500
C	-5.43047400	-0.98444200	-0.46068500
C	3.37842100	1.05236500	-0.03421500
C	4.74645200	1.22814000	-0.20392900
C	5.58641900	0.11979600	-0.32198400
C	5.05481100	-1.17092700	-0.26894400
C	3.68928700	-1.35587300	-0.09978600
O	-4.18599800	-1.67366900	-0.19821800
O	-1.56993600	2.35091600	0.09177400
C	2.83544800	-0.24391300	0.01827300
C	0.64244800	-1.58459300	0.17013400
C	1.40640600	-0.43316900	0.19042200
C	-1.91178100	-1.83179000	0.14245500
C	-0.70272500	-1.20413700	0.32645100
C	-0.66678700	0.24848900	0.68581100
C	-1.80883500	1.06051000	0.15969000
C	-2.99466500	0.41512400	-0.05884000
C	-3.05359900	-1.01692900	-0.01129400
O	0.66336300	0.68536900	0.38981700
H	-3.42870000	3.20369400	0.51026900
H	-3.04262700	3.01424500	-1.23448200
H	-2.19441700	4.24532200	-0.25598100
H	-5.36870900	-0.43570600	-1.40089100
H	-5.67691100	-0.31809600	0.36622200
H	-6.17382200	-1.77239400	-0.53847500
H	2.72671100	1.91135600	0.05553000
H	5.15856000	2.22917800	-0.24513000
H	6.65287800	0.26010500	-0.45395700
H	5.70653600	-2.03166400	-0.35722800
H	3.28921700	-2.36085200	-0.05354300
H	1.00462200	-2.57963300	-0.02859800
H	-2.00097700	-2.89691100	-0.02182100
H	-0.77190100	0.31190800	1.78201400
H	-3.88295900	0.97526100	-0.30272900

α-keto cation, solvent=water

1 1

C	-2.98009300	0.46074200	0.06034300
C	-1.83247600	1.18023600	-0.34168500
C	-0.63057400	0.51485200	-0.51102000
C	-0.57970800	-0.89064500	-0.27923500
C	-1.74646700	-1.61114100	0.11671400
C	-2.94004200	-0.92785900	0.29128300
C	0.60212900	-1.60077500	-0.40118500
C	1.88896000	-1.07912600	-0.97012900
C	2.91120700	-0.41180200	-0.17311100
C	4.09960900	0.01085100	-0.79498300
C	5.08492600	0.63119600	-0.04255400
C	4.89558600	0.82831300	1.32991800
C	3.72046800	0.40673600	1.95297600
C	2.72515000	-0.21092400	1.20436500
O	1.94387300	-1.33806300	-2.16522100
O	-1.82961100	2.50545000	-0.57544900
O	-4.00483700	-1.65698900	0.67405800
C	-5.27756300	-1.01446100	0.85858300
C	-3.04230200	3.26227300	-0.41988700
H	-3.91198600	0.99041500	0.19185700
H	0.24643100	1.06698200	-0.82048400
H	-1.70082900	-2.67885800	0.28456000
H	0.59298800	-2.67290800	-0.21934900
H	4.23147400	-0.15388400	-1.85721500
H	6.00144700	0.96127000	-0.51599800
H	5.66957500	1.31193700	1.91443300
H	3.58156800	0.56085900	3.01575200
H	1.81253900	-0.53624900	1.69087000
H	-5.95788700	-1.80759400	1.15716500
H	-5.22232700	-0.26171100	1.64835400
H	-5.62482400	-0.56492800	-0.07460700
H	-2.77331200	4.28615500	-0.66564800
H	-3.81213000	2.90966600	-1.10991700
H	-3.40099000	3.21486700	0.61076000
